# Extracts

**Published:** 1883-12

**Authors:** 


ATLANTA
MEDICAL REGISTER.

VOL. III.] DECEMBER, 1883. [No. 3

### Extracts.

CLINICAL REMARKS ON DYSMENORRHŒA AND STERILITY. —(*C. D. Palmer, M.D., in Cincinnati Lancet and Clinic*).— Attention is called to the following group of cases, as illustrative of several interesting points in connection with the subject of dysmenorrhœa and sterility; more especially as regards the pathology and treatment. In the minds of physicians in general there are few subjects about which there exists so much obscurity. At the same time, in no gynecological affection is a determination of the underlying cause more necessary. These few cases are cited then, as examples of many cases met with in this dispensary.

Case I. Mrs. B., æt. 33, married sixteen years. Has never been pregnant. States that she has suffered from dysmenorrhœa at every menstrual period since its inception, which occurred at twelve. Menses regular, appearing once in every twenty-eight days and lasting four or five days, but rather scant.

Physical examination reveals an enlarged and indurated uterus, which is tender to the touch; also, evidences of chronic cervical endometritis, such as erosion of the cervix, and a patulous os, from which hangs the characteristic cervical discharge. General health is good.

*Remarks.*—The examination has revealed these facts in regard to the local lesions, but what is the nature of the painful menstruation? To a superficial observer it might appear to be either congestive or inflammatory, but such is not the case; for it is more than probable that the local organic lesions are secondary *to* and the result *of* the long continued sterility and persistent dysmenorrhœa. It is not of the obstructive variety, for there is no difficulty in passing a good sized sound. Furthermore, the pain continues, though the flow is scant; and the patient states that the pain is rather diminished when the flow is free. Two factors in the case lead us to the final conclusion that we have to deal with a case of neuralgic dysmenorrhœa. First, she has been subject to the disease since puberty. Second, the pain commences on the day before the flow, and gradually increases in severity; reaching its maximum on the second day, it rapidly disappears.

The history of the case was probably as follows: A primary neuralgic dysmenorrhœa, which, like other forms of nervous irritation long continued, has induced a chronic congestion of the uterus. This in its turn has been followed by the cervical and some corporeal inflammation, upon which the sterility is consequent. Primary dysmenorrhœa, coming on at puberty, is not associated with organic lesion. The uterus is frequently undeveloped but not inflamed at this time of life.

*Treatment.*—The first indication is to overcome the local lesions. This is to be accomplished by means of the hot vaginal douche, twice daily, and by the use of tampons, wet with tanno-glycerine, thrice weekly. Local depletion by puncturing is also useful and will be utilized here. About one drachm of blood is drawn off for each puncture. Now, you will notice the improved appearance of the cervix almost immediately. In the way of general treatment, the patient will receive potassium iodide—seven and one-half grains ter in die—and at the time of menstrual period, tincture cimicifuga will be administered.

As regards prognosis, there is a reasonable prospect of relieving the dysmenorrhœa in a few months; but as to the sterility, little can be promised. For the relief of the latter, as well as the former, there is nothing more to be done than a persistent treatment to remove what is tangible, and put the uterus in the best possible condition. The cause of the dysmenorrhœa is more clear than that of the sterility. We are to remember, too, that fertility is a more complex process, and the prognosis of sterility is *always* a matter of uncertainty. Even when the cause is apparent in the *wife*, it is well to examine the *husband*. For the *real* cause is sometimes with him, though not suspected.

Case II. Mrs. C., æt. 31, married three years, sterile. General health poor and general system ill developed. Menstruation is very irregular, appearing variously from three to five weeks and lasting seven or eight days. There is uterine leucorrhœa.

Local examination reveals a small pointed cervix with a so-called "pin-hole" os. Also, decided ante-flexion; the angle at the upper portion of the cervix being about ninety degrees. The body of the uterus is tender and slightly enlarged.

*Remarks.* — Patients with these physical conditions almost always suffer from dysmenorrhœa and are sterile. The interesting feature of the case, however, is the fact that, although there is true cervical stenosis combined with genital flexion, the dysmenorrhœa of which she complains is *not* obstructive. The patient has no distinct *uterine* pain, but complains rather of pain in the right ovarian region, most marked *before* the flow. During the menstrual period, when the flow is comparatively abundant, she is almost free from any distress. The local conditions of the cervix are congenital, having arisen at the second period of developement; but the condition of the body of the uterus, *i. e.*, its tenderness, enlargement, etc., are secondary, and is the result of impeded venous flux, owing to flexion and the imperfect drainage of the uterus on account of the stenosed canal. The ovarian pain may be and probably *is*, the result of sympathetic irritation. The ovary cannot be felt to be enlarged. Primarily, the sterility was obstructive, and why? Because the stenosis of the canal and the angle of flexion offered an impediment to the ingress of the spermatic fluid. At the present time, however, another cause for the sterility might be added. Aside from these congenital difficulties, the acrid discharges from the inflamed mucous membrane would probably be fatal to the spermatozoa, were their entrance into the canal a possibility. Unless something is done for this patient, she will gradually grow worse from year to year; the ovarian pain and endometritis increasing steadily. The patient will be apt to become, not only permanently sterile, but a great sufferer from incurable dysmenorrhœa, and finally a confirmed invalid. There are conditions here which nature cannot relieve.

The question now presents itself as to what is best to be done. Our attention must first be directed to the uterine tenderness, for as long as it exists, operative interference is wholly out of the question. This indication is to be met by the daily use of the hot vaginal douche, as in the preceding case. Also by tampons of glycerine so constructed and so placed—high up in the anterior cul-de-sac, against the body of the uterus—that they serve the double purpose of relieving congestion (by drainage through the exosmosic effect of the glycerine), and mechanically lifting up and temporarily straightening the uterus. After having accomplished this, the congenital stenosis of the os and the seat of flexion are to be overcome by incision. Emmet's method of incising the posterior lip is the best. We are greatly indebted to this distinguished authority for pointing out the differences in the seat, kind, and degree of flexion in the cervix and in the body. This flexion is in the cervix, and by incision we can improve the canal, which we could not so easily do, were it at the junction of the body and cervix. It should be made into and through the posterior lip nearly to the vaginal junction. As the posterior cervical lip, owing to the seat and kind of flexion, is longer than the anterior, this operation is also indicated. If this incision does not reach the angle, another incision can be extended further by the knife along the anterior wall. After the operation care must be taken to keep the canal open until cicatrization has taken place. Bilateral incision could do no good and might do harm, if made too deeply through the cervical wall; for it would have left the canal gaping, patulous, and the cervical mucous membrane liable to oversion. In due time, after the tenderness has been relieved and the uterus has been given time to be in a measure straightened up under the use of the tampon, we will recommend such a procednre. The sound might be used to straighten the uterus, but as it is liable to do considerable mischief through mechanical force, setting up serious inflammations, both in the nterus and in the surrounding cellular tissue, the former mode is preferable.

As far as the ovarian pain is concerned, the best we can do is to administer the bromides during the period in which the patient usually suffers.

If tonics are given they should be vegetable and not chalybeate.

Here also we can confidently expect relief to be obtained of the dysmenorrhœa and the plan of treatment to which I have referred, if it does not overcome the sterility (which is probable), will have a good prospect of staying the progress of the local trouble, otherwise sure to increase until the menopause brings about relief.

If such patients were put under proper treatment soon enough, it is my belief that many more might be made fertile.

I wish you to observe that in these two cases of dysmenorrhœa, with different underlying or associated conditions, we have very good evidence of the fact that *dysmenorrhœa is rarely obstructive.*

The first has dysmenorrhœa, and has had it at every menstrual period since puberty, yet the canal of the uterus is free.

In the second case, although the external os is small ("pin-hole"), and the canal really obstructed by decided flexion, there is no uterine dysmenorrhœa. The pain complained of is not *during* but *preceding* the flow. Here, then, there is seeming and real obstruction and yet no dysmenorrhœa.

Case III. The next case might well be classed as one of artificial or acquired sterility, but bears upon the subject of dysmenorrhœa only as being another instance in which stenosis is not accompanied by obstructive dysmenorrhœa;

Beside the above, it presents several features of particular interest.

The patient, a Mrs. M——, æt. 33, married, with one child (born eighteen years ago), came to the clinic July 3, 1882, complaining of pain in the back and groin, nausea and vomiting (especially after eating), and obstinate constipation. There was much general nervousness. The most serious feature of the case was her statement that her menses appeared once every fourteen days and lasted the same length of time, there being practically a continuous flow. The quantity was not especially excessive at any time, but the continued discharge of blood had given much uneasiness of mind, as well as being a great physical drain. There was also troublesome lucorrhœa and pruritis pudendi. She had suffered from dysmenorrhœa, but as this fact was only elicited on close investigation, it could not have been very severe. The menorrhagia and metrorrhagia were of five or six months' duration.

Local examination showed the uterus to be movable, retroverted to the first degree, and slightly prolapsed, being about two and one-half inches from the vulvar orifice. The body of the uterus was enlarged, and extremely tender to the touch. The cervix was enlarged and indurated, the external os being small and the canal stenosed. Patient stated that at some previous time, while under treatment, caustics had been applied to the "neck of the womb." Here was an interesting condition, with a view to determine the cause of the hemorrhage. No evidence of an abortion with retained products of conception, nor seemingly of malignant disease, and no tumor in the cavity of the uterus was suspected, because the os and canal were small.

Measurement by the sound showed the uterus enlarged, but only three and one-half inches. A diseased endometrium of the upper cavity was suspected. Cauterization for cervical erosion, having been injudiciously continued, had produced stricture of the canal, and the obstruction to the menstrual flow had led to or aggravated an existing endometritis, and finally a fungoid degeneration of the corporeal endometrium had subsequently developed.

This *presumptive* was followed by *positive* evidence, when, at a subsequent sitting, some of the fungosities were removed by the use of a small dull wire curette, which was introduced with considerable difficulty through the cervical canal, but which swept freely around the cavity of the body. The operation was followed by complete though temporary relief from the metrorrhagic symptoms. October 21st patient returned, stating that up to that time she had menstruated regularly as to time and quantity, but her menorrhagia had now returned. Curette was again used with good result, this time more permanently.

During the preceding month (September, 1883,) she again returned, and upon examination it was concluded that, although the curette might be used to an advantage, it would fail to reach the underlaying cause—the strictured canal. Stenosis acts here like a stricture of the urethra in causing dilatation of the bladder and consequent cystitis. Hence, to overcome the difficulty, we must strike at the original link in the chain of disease. Dilatation by sounds and tents would open up the canal, but for a short time, and the relief would be only temporary. Incision holds out more permanent benefit, and it has been employed (September 28th) in the following manner: The patient having been placed on her back, with the thighs flexed upon the abdomen and the legs upon the thighs—in Sims' position—the uterus exposed by two specula, and steadied by a tenaculum, a small narrow-bladed knife was introduced into the uterus and the posterior cervical lip incised, beginning at the internal os and gradually going deeper, from within outward, until the os tince was reached. The anterior lip was then incised in the same manner. The lines of incision were made anterior and posterior rather than lateral, because at these points the stricture seemed most defined. At the same sitting the dull wire curette was used as before, and the cavity of the body carefully but thoroughly scraped. Patient was directed to remain quiet in bed for five or six days. No untoward symptoms have as yet developed, and it remains to be seen whether or no the treatment will prove effectual.

While attending the clinic the patient has also received some accessory treatment, as liquor potassii arsenitis as a stomach and nervous tonic, having at the same time a beneficial effect upon the menstrual disorder. The bromides were given occasionally to allay the extreme nervousness. When constipated she was given that little pill so often prescribed here: Podophyllin, ext belladonna, ext. nucis vomicæ aa gr. ⅛.

*Remarks.*—This case has been an exceedingly interesting one. First, as to the diagnosis of the cause of her menorrhagia and metrorrhagia, which, after excluding all else, was clearly proven to be a fungoid endometrium; second, as showing the ill effects of caustics, too frequently injudiciously applied. We do not so often see these ill effects of late as in former years, because, fortunately for suffering women, cauterization of the uterus is becoming unfashionable. Caustics are useful topical remedies to the diseased uterus, but should be comparatively seldom used. And even when indicated, they should be employed with caution, for fear of inducing undue contraction of the cervical canal, causing, eventually, as in this case, chronic uterine catarrh, sterility, etc., a condition of affairs which, with the general and logical reflex symptoms, is much worse than the original mischief. This case also illustrates the benefits of the curette—a very valuable little instrument, safe when properly used, and attended in certain cases with very brilliant results.

All three cases are good illustrations of the fact that we must not look to symptoms alone, but search for the pathological conditions behind, producing the symptoms. Treatment of pain at the menstrual period with *anodynes* can be attended with no permanent good.

Strike at the *root* of the trouble, remove it, and the branches will be cared for also.

---

Cow's Milk for Infants.—At the annual meeting of the Massachusetts Medical Society in 1882, Dr. J. P. Lynde, of Athol, delivered an address upon "Infantile

Mortality, its Causes and Prevention." This address is replete with sound advice, and, although intended solely for professional reading, it should not be restricted within so narrow limits; it should be read by every mother and nurse in the country who have children and infants in charge. That portion of the address devoted to the consideration of foods for infants is especially sound and sensible, and we present copious extracts from this part of the paper.

The prevailing custom with physicians and nurses is to use cow's milk diluted with one, two or three parts of water, with the addition of a little salt and sugar. Some prefer milk, water and cream in variable proportions, or the use of some one of the many patent commercial foods that have recently been invented. Others rely upon thin gruels of arrow-root, corn-starch, gelatine, barley, oat-meal or wheat, either pure or enriched with milk. Such methods of artificial alimentation are recommended in all standard works on medicine and by almost all teachers.

The books abound with learned arguments to prove that it is dangerous to administer undiluted cow's milk to an infant, and minute directions are given how to dilute, adulterate and render it more acceptable and safe.

The chemist has been asked to determine by the methods of his subtle art the constituent elements, and the difference between human and cow's milk; and he finds proportionally more sugar in human, and more caseine in cow's milk, with less important variation in the fatty, saline and animal elements. He also finds that the caseine of human and that of cow's milk differs in its physical characteristics when acted upon by reagents; that of the cow forming a more tough, firm, indigestible curd.

Different analyses give variable results in all particulars, as might be expected; for it is apparent that the composition of any milk, human or animal, will be modified by the season of the year, the quality of the food and drink, the state of health, the time since parturition, individual and race peculiarities, and many other inflnences. Again, milk has certain volatile elements, and it may be polluted with septic germs, or the infection agent of severe or deadly disease, which no art of the chemist can detect. So that, whatever may be the results of analysis showing differences in the grosser constituents, they are not of sufficient practical importance from which to deduce the conclusion that it is dangerous to feed infants with undiluted cow's milk, and that they cannot be safely and successfully raised in this manner. . . .

The artificial substitutes for human milk that have been mentioned have certainly been most thoroughly tried for many years, and the results have not been very flattering to the scientific acumen of the inventors. Thousands of helpless, beautiful children have been yearly starved to death, poisoned and made sick by the use of these unsuitable, indigestible foods. . . .

All theories and methods of administration, to be accepted as fixed, must successfully stand the great crucial test of science—that of observation and experience. What testimony, then, can we secure from observation and experience?

In 1868 Dr. Stephen Rodgers, of New York, published an article in the *Medical Record*, recommending undiluted cow's milk for infants, after nine years' experience, and its use in bringing up three of his own children.

Dr. Hiram Corson, in an address before the State Medical Society of Pennsylvania, on food for infants, some fifteen years ago, after observations extending over a period of more than thirty years, declares: "I feel quite certain that it is almost as easy to raise children by hand, if they have an abundant supply of good, undiluted cow's milk, as it is by the breast."

His experience confirms his opinion "that thousands of children who die annually of diarrhœal diseases die for want of food. They are really starved to death." He says to the profession, "And we are not blameless." . . .

A discussion on the question of nourishment, in the pediatric section of the fifty-fourth meeting of German naturalists and physicians, took place at Salsburg, September 19, 1881. A commission had been previously appointed to prepare papers and investigate this important subject. A circular was prepared and widely distributed, directing the discussion to two points:

"First—Substitution of natural, unadulterated animal (cow's) milk for human milk, and its production.

"Second—Substitution of artificial foods, with or without milk, for the natural milk; their nature and value."

After earnest discussion for two days, the conclusions reached in regard to artificial foods were expressed by Dr. Soltmann as follows: "Now and evermore it is unanimously agreed that these preparations *can in no way be substituted for mother's milk, and, as exclusive foods during the first year, are to be entirely and completely rejected.*"

In regard to cow's milk he said: "Therefore, we now stand at this point: Cow's milk is the only substitute for mother's milk. Our whole endeavor must be to procure and use this in the best way. This standpoint is a long step in advance on the broad field of work before us." As presiding officer, he closed the discussion thus: "Let us hold fast, gentlemen, to what we have already gained. Let us attempt in the future to put it in the power of even the poorest to obtain good, pure milk for his children. United work on the part of doctor, experimental pathologist, physiological chemist, and land-owner, can alone fill up the gaps in our knowledge of dietetics."

In artificial feeding we should carefully observe the methods and follow the indications of Nature; therefore, we would satisfy the infant's thirst by allowing it to draw water from a tumbler slowly through the nurse-tube. Some children will require considerable; others but little. Not being an aliment, it does not excite the secretion of the gastric juices. Then in a few moments allow the child *all the fresh, undiluted cow's milk it will take*, warmed to the temperature of the blood, only being sure that it is slowly ingested, just as Nature supplies it when nursed from the mother's breast.

Some will ask, "Why not mix the water and milk in the bottle, instead of in the stomach? What is the difference in result?"

Milk and water is neither the one nor the other. It is extended, adulterated, weakened milk, and, when thus administered, more bulk is required for a given amount of nourishment; the stomach is over-distended, and the gastric juice so diluted as to weaken its digestive power; besides, the water and milk separately administered mix but little in the stomach, the water is so rapidly absorbed. By this method we do closely imitate Nature, and in a great measure—yes, almost entirely—overcome the difference in quality and digestibility of the caseine between human and cow's milk. . . .

The cow should be selected with great care. Milk from different breeds, and from cows of the same breed, varies widely in its essential elements. That from the Jerseys and Alderneys is excessively rich in cream. The Ayrshire and grade cow should be preferred, as they furnish a fluid more nearly resembling human milk. A very fat baby is not to be desired. The quality of milk is influenced by the season of the year, and the food, air, water and care which the cow receives, and the state of her health. For obvious reasons we would avoid a diseased, old or farrow cow—one fed on turnips, onions, cabbage, slops, garbage and coarse, our swamp grasses, supplied with water from a stagnant pool or polluted well, or kept in a damp, filthy, ill-ventilated stable—and select a young, healthy Ayrshire, or grade cow, that has recently had a calf; one that has good, sweet, upland pasturage, with pure spring water to drink, and plenty of salt; one that is sheltered in a clean, well-ventilated stable; one that has good care, that is kept quiet and gentle, not worried by dogs or boys. We would examine the milk with a test-tube, note its specific gravity, its color, smell and taste, the percentage of cream, its reaction to test-paper, and, if acid, reject it. We must use our eyes, our nose, our taste, and secure pure, rich milk, milked morning and evening from a clean cow into a clean pail; keep it in a clean bowl, covered with a clean napkin, in a clean closet, away from the pantry where food is stored. It will rapidly absorb odors, dust and septic germs, and in hot weather will quickly ferment, putrefy and spoil, unless cared for, and thus become the vehicle or cause of severe or perhaps deadly disease. It is the most sensitive and easily spoiled of all animal foods.

Having secured good milk, we would procure a suitable nurse-tube and bottle; one with a screw in the stopper is desirable to regulate the flow. We must be sure that the child draws the milk slowly, just as in nursing, for, if poured into the stomach faster than the gastric juices can be secreted to mix with it, it may form a hard mass of curd, distress the child, and perhaps cause a convulsion.

The nurse-tube and bottle should be kept scrupulously clean and sweet by rinsing in a weak soda-water immediately after using. Perhaps, after all our care, the milk may not be acceptable to the delicate stomach; then render it more alkaline by adding a suitable quantity of bicarbonate of soda or potassa, or the phosphate of soda; or try the milk of another cow. The trouble may be due to bacteria or microdemes; then sterilize the milk, as advised by Tyndall, by subjecting it to the heat of 150° for a few minutes.

I never boil milk for a child; it changes its specific gravity and other characteristics. Salt should not be forgotten.

We cannot manage all children by one precise rnle. Rules and methods must be elastic, and varied to meet individual idiosyncrasies and peculiar conditions.—*Popular Science News.*

---

NATIONAL TRAITS IN SCIENCE.—An article in *Science*, an illustrated weekly journal, recently established at Cambridge, Mass., discusses the peculiarities and points of excellence and inferiority of the different nationalities, which make pretence to distinction in the direction of science. While the article treats of no particular division of science, its remarks are quite applicable to medicine in its scientific features as it is cultivated in the different countries referred to. There are at present three principal currents of scientific work—German, English and French. In no other nationality has the current attained sufficient volume or individuality to warrant its classification as other than a tributary. The importance of these currents is in the order in which they are named, and

German influence is now predominant over all the scientific world, students from all countries, excepting France alone, now flock to Germany and bring back with them the German methods, which are in this way blended with such as are developed *de novo* in their own country, and thus the sway of Germany over western thought becomes potent and wide spread.

The distinctive qualities of the German scientist is that he is an investigator. This he is obliged to be, as he wins his position in the hierarchy of learning by the original researches which he carries out. This necessity imposed on him, carries with it the additional necessity of a thorough familiariiy of all that has gone before in the way of additions to the general funds of scientific knowledge; in order to know what is new the investigator must necessarily be familiar with what is old. It follows, therefore, that the German scientist is, perhaps, more fully abreast of the knowledge of the day than his confreres in any other nation, being fully posted as to the status of his department, and often displays a singularly just and keen appreciation of what problems are for the moment best worth studying as being open for solution and leading to something further, or else filling the gap which is left. It is sad to think how much scientific work is wasted because the labor is not wisely directed. In strong contrast, however, with the German thoroughness is his literary style. German scientific writers are notoriously very awkward and slovenly in their literary composition. That conciseness, clearness and elegance of expression which obtains in England, for instance, is unknown in Germany, where an easier plan of great diffuseness seems to prevail. There are, of course, celebrated exceptions to this rule, but they are individual. The nation regards itself as having a decidedly philosophical tendency, meaning a facility at taking broad and profound views of the known; but its profundity is mysticism, its breadth, is vagueness. Germany has contributed very little to the generalizations of science. She has produced no Linne, Darwin, Lyell, Lavoisier or Descartes, each of whom bequeathed to posterity a new realm of knowledge, although she has given to the world grand results by the accumulated achievements of her investigators. The German, moreover, has a very imperfect sense of humor, and this constitutes another serious obstacle to his progress. He is thus cut off from the perception of some absurdity which cannot be explained to him. It were easier to explain a shadow to the sun who always sees the lighted side. To state the whole epigrammatically, German science is the professional investigation of detail, slowly attaining generalization.

English science is the opposite of the German. In fact, the professional investigator has hardly been a recognized character in the English social organization. Until recently he has barely acknowledged even by the universities, who sought instructors who knew and could teach, who might investigate and discover in a subsidiary, and, as it were, unofficial way. The English investigator as he has existed, has been disconnected from the universities and colleges, and the writings of this class show the effects of this separation by a lack of thoroughness and imperfect acquaintance with investigations of others, and a grasp the essential part of the problem. In brief, their writings appear behind hand and superficial, yet amid these poorer productions are to be found a right goodly number of scientific articles that would do credit to any nation. While the English lack in thoroughness of investigation they are trained writers. Their articles excel the Germans in literary merit, being rarely slovenly, either in arrangements or style, and are seldom wearisome from sheer diffuseness. The noteworthy characteristic of the English scientist is virtually in generalization. This is with the English the outcome of individual endowments, a single master attaining a broad conclusion —a process of individual effort quite unlike the German Democratic method of generalizing by the accumulations of many. The English and the Scotch may be said to be Greeks of modern philosophy.

French science is decidedly provincial. The French have lagged far behind the great movement of recent years. Consider only how backward they have been in the comprehension and acceptance of the Darwinian theory, and remember, too, that it were wiser to take the main spring from a watch than to eliminate evolution from biology. The literary part of the French scientific writings is excellent. The keen, artistic sense of the nation displays itself here, but it also deludes them into presenting and mounting a survey of a greater field than is demanded by the actual discoveries they report. To satisfy this yearning for artistic completeness, elaborate and tedious disquisitions and hackneyed principles and facts long known are interpolated, and even worse, may be, when the imagination helps to create the completeness. Most scientific men harbor a little distrust of French work. This sentiment is further fostered by the almost systematic neglect of German research on the part of the French. The French stay at home. They used to travel abroad much, and yet there are a few savants in France who are esteemed throughout the scientific world.

America's contributions to science are by no means very extensive, or very often important, that is as compared with the great volume of German production, in which comparison they seem almost insignificant. We have never duly fostered research, for we have bestowed upon it neither the proper time nor office. There are, we suppose, at least six thousand "professors" in the United States. Are there one hundred and fifty of them active investigators? The time seems remote when every American Professor will be expected to be also an investigator, or to contribute aught to the common fund, but among us is a little band of men who have before them the model of Germany, and who are earnestly working for the intellectual elevation of their country. The first object is necessary to render research more important in public estimation, and so to smooth the way for a corpse of professional investigators. Every thoughtful person must wish success to the attempt.

---

Substitute for Mercurial Ointment.—A French physician, Dr. R. Vizier, recommends four or five per cent. solution of corrosive sublimate in glycerine in place of mercurial ointment for parasites of the skin. Glycerine is not absorbed by the skin, and to a great extent prevents the absorption of medicines. Therefore, on account of its greater cleanliness and greater security from the absorption of mercurials, this solution is to be preferred to blue ointment.—*Pacific Medical and Surgical Journal.*

---

Salicylic Acid in Typhoid Fever.—M. Vulpain (Medical Press) gives his preference to salicylic acid, as compared with the sulphate of quinine in typhoid fever. He holds it to be not only antithermic, but also antipyretic, although we are unable to fully understand the difference between those two terms which is implied by his use of them. He has never found it to produce any deleterious action on the heart, and has remarked the absence of bad sores in cases in which it has been employed. We presume he has reference to large doses of salicylic acid in those cases, for we think the experience of practitioners is very unanimous on not only the uselessness, but possitive injuriousness of small doses, at least of the sulphate of quinine in typhoid fever. This drug is permissible in this disease only as an antipyretic, for which it must be given in very large doses. In our experience, however, this is beneficial rather in preventing the rise of temperature after its reduction by means of cold baths, than in directly reducing it *per se.*

---

Human Obesity.—Last week the death was recorded of the "fattest woman in the world," a member and special curiosity of a traveling circus company in America, who appears to have been smothered in her bed by rolling upon her face, she being unable to turn upon her back without assistance. Miss Conley, the adipose prodigy in question, though probably the most enormous of her sex, weighing, as she is stated to have done, 497 pounds, fell far short of the bulk of the famous Daniel Lambert, who died in 1809, at Stamford, at the age of 40. Lambert weighed no less than 52 stones 11 pounds, that is 739 pounds, or close upon half as much again as the American lady. He was 5 feet 11 inches in height, measured 9 feet 4 inches round his body, and 3 feet 1 inch round the leg. It is said that he never drank any alcoholic beverage, and that he usually slept less than eight hours a day.

---

The Use of the Bromide Salts for Abdominal Neuroses.—There is so strong a bond of therapeutic association between the bromides and the neurotic troubles of the head and chest that we are apt to forget how useful the same drugs may be for sundry disturbances of the digestive organs; and yet all the physical analogies of the subject would lend support to this doctrine. No one claims for the potassic and sodic bromides that they can clear away heterologous exudation, and mend damaged textures. But those of us who are still old-fashioned enough to believe in "functional derangements," or dynamic force temporarily perverted, can easily understand that there are certain aberrations of the cerebro-spinal system, which, being of the same kind wherever they are situated, may be expected to yield to the same medicines.

For an elderly widow lady, tormented rather often with "emotional diarrhœa," I prescribed a few years ago some ordinary astringent remedies, with minute doses of opium, to be taken according to her needs. But for another malady, sleeplessness, I gave occasionally moderate quantities of bromide of potassium. She discovered, however, that the latter remedy did her diarrhœa more good than anything else, and that, whenever it was taken at bed-time, the next day passed without any alvine looseness

Fourteen years ago Dr. Waring Curran recommended potassic bromide for the vomiting of pregnancy; but its real value could not be determined, as other things were combined with it (*Medical Press and Circular*, July 14, 1869). But I have given the medicine in the pure form, and simply dissolved in water, and never without marked, though perhaps temporary, success.

The distant echoes of cholera justify us in recalling some important observations by the late Dr. James Begbie, who spoke of bromide of potassium as able to strip that dread disease of some of its terrors (*Edinburgh Medical Journal*, December, 1866). He gave it in the earlier stage of the collapse, and in quantities of twenty or thirty grains, at hourly, or even half-hourly, intervals; and he records the cessation of vomiting, the arrest of cramp, and the speedy return of warmth and color to the previously cold and livid surface. He tells us that the medicine was tried fairly, both in the Leith and Edinburgh Cholera Hospitals, and that its use in both institutions did not disappoint expectations. It is good to feel better fortified against the most painful and mortal of all abdominal neuroses.

Lastly, I may glance at the use of the bromides in the treatment of saccharine diabetes. Here again Dr. Begbie started a line of therapeutic inquiry which has been successfully worked by other practitioners; and at this moment I have under my care a lady, between fifty and sixty years of age, whose special diabetic symptoms are clearly kept much in abeyance by a large dose of bromide of ammonium every night. Would this illustrate what has been called the "alterative and absorbent effects" of the bromides on the liver?—*British Medical Journal.*

